# Supplementary material for: Resolving the full spectrum of human genome variation using Linked-Reads
Source: Genome Res. 2019 Apr;29(4):635–45. doi: 10.1101/gr.234443.118 (PMC6442396; doi:10.1101/gr.234443.118)
Supplement: Supplemental Material [file supp_gr.234443.118_Supplemental_longranger-2.2.zip › longranger-2.2.tar.bz2!/lr2.2-public/lib/stan/cmdstan/stan/doc/api/html/classstan_1_1io_1_1dump-members.html]

Stan: Member List


|  |  |
| --- | --- |
|  | Stan  2.5.0  probability, sampling & optimization |


- Main Page
- Related Pages
- Namespaces
- Classes
- Files

- Class List
- Class Index
- Class Hierarchy
- Class Members

All Classes Namespaces Files Functions Variables Typedefs Enumerations Enumerator Friends Macros Pages

- stan
- io
- dump

stan::io::dump Member List

This is the complete list of members for stan::io::dump, including all inherited members.

|  |  |  |
| --- | --- | --- |
| add\_vec(std::stringstream &msg, const std::vector< size\_t > &dims) const | stan::io::var\_context | inline |
| contains\_i(const std::string &name) const | stan::io::dump | inlinevirtual |
| contains\_r(const std::string &name) const | stan::io::dump | inlinevirtual |
| dims\_i(const std::string &name) const | stan::io::dump | inlinevirtual |
| dims\_r(const std::string &name) const | stan::io::dump | inlinevirtual |
| dump(std::istream &in) | stan::io::dump | inline |
| names\_i(std::vector< std::string > &names) const | stan::io::dump | inlinevirtual |
| names\_r(std::vector< std::string > &names) const | stan::io::dump | inlinevirtual |
| remove(const std::string &name) | stan::io::dump | inline |
| to\_vec() | stan::io::var\_context | inlinestatic |
| to\_vec(size\_t n1) | stan::io::var\_context | inlinestatic |
| to\_vec(size\_t n1, size\_t n2) | stan::io::var\_context | inlinestatic |
| to\_vec(size\_t n1, size\_t n2, size\_t n3) | stan::io::var\_context | inlinestatic |
| to\_vec(size\_t n1, size\_t n2, size\_t n3, size\_t n4) | stan::io::var\_context | inlinestatic |
| to\_vec(size\_t n1, size\_t n2, size\_t n3, size\_t n4, size\_t n5) | stan::io::var\_context | inlinestatic |
| to\_vec(size\_t n1, size\_t n2, size\_t n3, size\_t n4, size\_t n5, size\_t n6) | stan::io::var\_context | inlinestatic |
| to\_vec(size\_t n1, size\_t n2, size\_t n3, size\_t n4, size\_t n5, size\_t n6, size\_t n7) | stan::io::var\_context | inlinestatic |
| to\_vec(size\_t n1, size\_t n2, size\_t n3, size\_t n4, size\_t n5, size\_t n6, size\_t n7, size\_t n8) | stan::io::var\_context | inlinestatic |
| validate\_dims(const std::string &stage, const std::string &name, const std::string &base\_type, const std::vector< size\_t > &dims\_declared) const | stan::io::var\_context | inline |
| vals\_i(const std::string &name) const | stan::io::dump | inlinevirtual |
| vals\_r(const std::string &name) const | stan::io::dump | inlinevirtual |
| ~var\_context() | stan::io::var\_context | inlinevirtual |


---

[ Stan Home Page ]

*© 2011–2014,
Stan Development Team.*
